# Chondrodysplasia Punctata: A Rare Entity Identified Incidentally

**DOI:** 10.7759/cureus.90894

**Published:** 2025-08-24

**Authors:** Laura Olarte Bermúdez, Valeria Noguera, Valeria Del Castillo, Santiago Pineda, Valentina Baez, Gustavo Triana, Hernan D Paez

**Affiliations:** 1 Radiology, Fundación Santa Fe de Bogotá, Bogotá, COL; 2 Medicine, Universidad El Bosque, Bogotá, COL; 3 Medicine, Universidad de Los Andes, Bogotá, COL; 4 Medicine Program, Universidad del Rosario, Bogotá, COL

**Keywords:** chest x-ray, child, chondrodysplasia punctata, congenital disorders, down syndrome

## Abstract

Chondrodysplasia punctata (CP) is a group of skeletal dysplasias characterized by abnormal endochondral ossification and epiphyseal stippling, often associated with genetic, metabolic, or teratogenic etiologies. We present a rare case of a four-year-old child in whom radiographic imaging revealed classic features of CP. The differential diagnosis of epiphyseal stippling includes a wide range of conditions, such as Zellweger spectrum disorders, Smith-Lemli-Opitz syndrome, and embryopathies from maternal autoimmune disease or warfarin exposure. Recognition of the stippled pattern in conjunction with rhizomelia and facial dysmorphisms on radiographs is critical for early diagnosis and multidisciplinary management. Although molecular testing may provide a definitive diagnosis, radiologic evaluation remains a cornerstone in identifying and characterizing these entities, especially when genetic testing is unavailable. This report highlights the pivotal role of imaging in diagnosing CP, particularly in atypical clinical contexts, and aims to contribute to the understanding of phenotypic variability and radiologic presentation in skeletal dysplasias.

## Introduction

Chondrodysplasia punctata (CP) is a group of rare congenital disorders characterized by abnormal calcifications (epiphyseal stippling) in the cartilage during fetal and neonatal development [1]. Although its exact incidence varies depending on the subtype, it affects both males and females, with manifestations visible as early as the prenatal period or at birth, with a prevalence of 2.4 million people in the United States presenting mutations that can cause CP [2,3]. CP is often associated with defects in endochondral ossification, often linked to abnormalities in specific metabolic pathways, such as peroxisomal metabolism or cholesterol biosynthesis [4]. In addition, more severe subtypes can lead to significant disabilities, including skeletal deformities, respiratory insufficiency, and multisystem complications that increase the risk of early mortality [5]. Clinically, it presents with punctate calcifications visible in radiologic studies, disproportionate dwarfism, joint contractures, nasal hypoplasia, and, in some cases, involvement of internal organs such as the heart and respiratory system [6]. The pathogenesis of CP is heterogeneous and may involve defects in peroxisomal metabolism, abnormalities in cholesterol biosynthesis, or disruptions in vitamin K-dependent pathways [1,6].

This entity is extremely rare, and the diagnostic challenge is posed by clinical manifestations and image resemblance to pathologies such as Zellweger spectrum disorders, Smith-Lemli-Opitz syndrome, warfarin embryopathy, and other skeletal dysplasias characterized by epiphyseal stippling. Currently, the literature highlights a significant gap in knowledge about this condition. Therefore, we will present a four-year-old child with CP associated with Down syndrome, presenting with severe nasal hypoplasia, joint contractures, and stippled calcifications identified on X-ray imaging. In this paper, we aim to emphasize the imaging findings and the presenting complication, given its rarity and potential relevance for clinical decision-making in similar cases of CP.

## Case presentation

**Figure 1:**
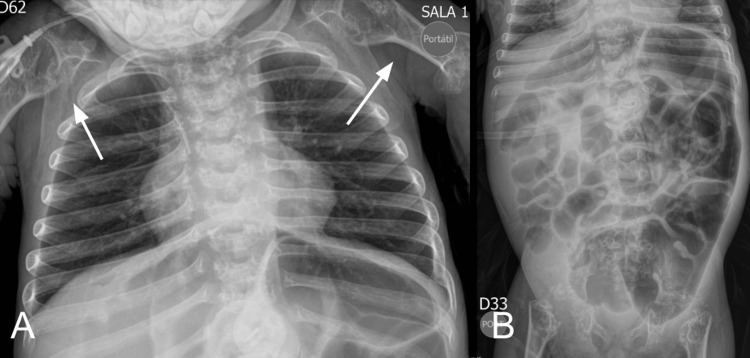
Chest X-ray (anteroposterior view) and abdominal X-ray (anteroposterior view) Chest X-ray (anteroposterior view) (A) shows characteristic punctuated sclerotic bone lesions in the epiphysis with multiple punctate, sclerotic cartilaginous calcifications distributed in all visualized osseous structures, particularly prominent at the epiphyses. There is rhizomelic shortening of the humeri, with evident reduction of the proximal segment length, associated with mild metaphyseal flaring of the distal humerus (white arrows). Cortical outlines are preserved, and no pathologic fractures are identified. The ribs demonstrate normal morphology, and no pulmonary parenchymal abnormalities, pleural effusion, or mediastinal enlargement are observed. Abdominal X-ray (anteroposterior view) (B) shows moderate gaseous dilatation of multiple bowel loops, without evidence of pneumatosis intestinalis, portal venous gas, or free intraperitoneal air. The osseous structures demonstrate diffuse punctate cartilaginous calcifications involving the pelvis, femora, vertebrae, and iliac bones. Both femora show significant rhizomelic shortening with mild metaphyseal flaring. The acetabular roofs are poorly developed bilaterally, suggestive of associated developmental dysplasia of the hips. Sacroiliac joints and pubic symphysis are preserved. The vertebral column demonstrates adequate alignment without congenital segmentation anomalies.

**Figure 2:**
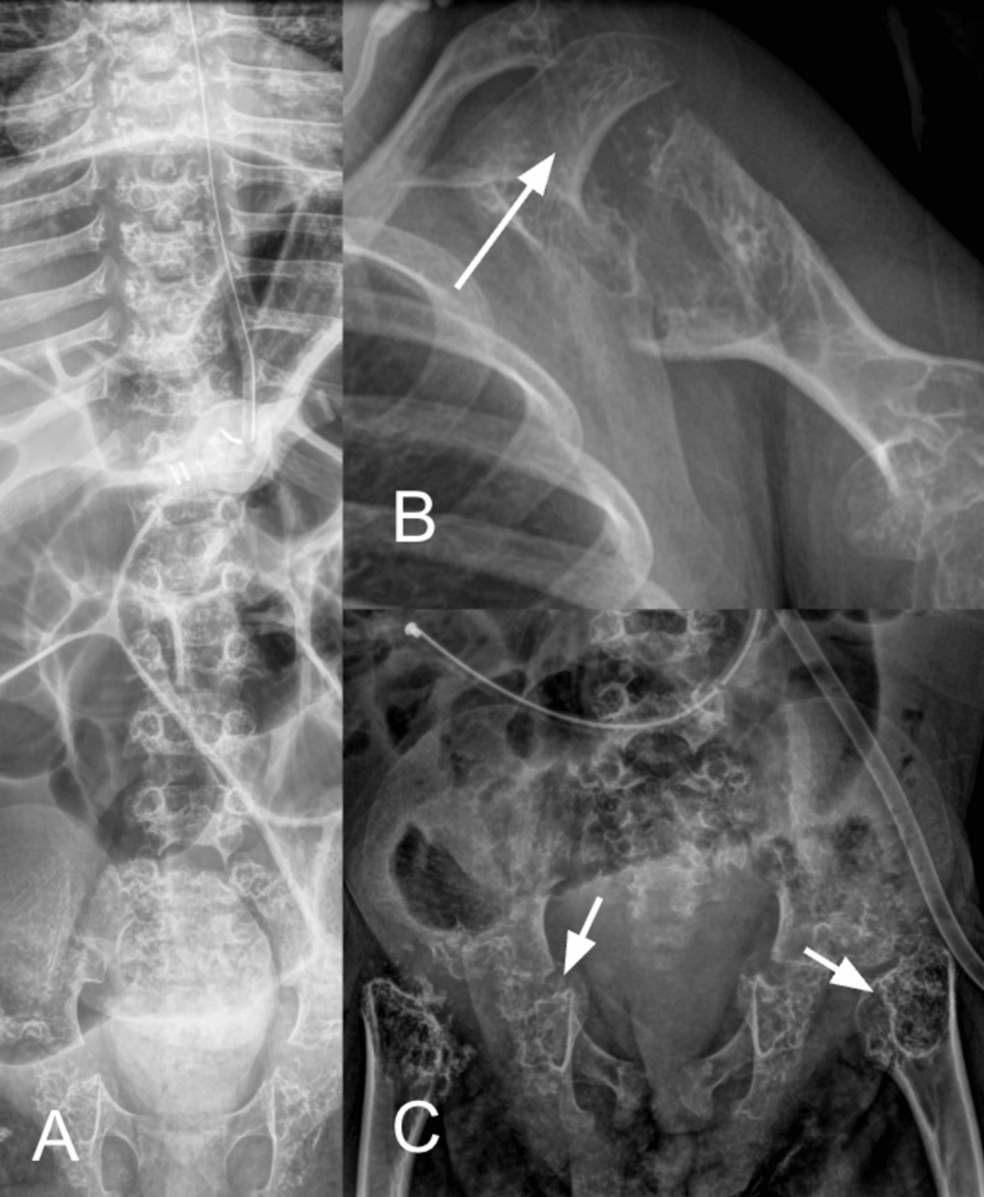
X-rays of the spine (A), left humerus (B), and pelvis (C) Characteristic magnified radiographs of the spine, left humerus, and pelvis show diffuse punctate cartilaginous calcifications involving all osseous structures (A–C). Multiple sclerotic lesions are noted at the epiphyses, associated with generalized rhizomelic shortening of the long bones. There is epiphyseal and metaphyseal widening, more evident in the proximal humerus (B, white arrow) and pelvis (C, white arrows). The vertebral bodies demonstrate punctate calcifications with preservation of height and alignment. Pelvic radiograph (C) further highlights irregular acetabular development with bilateral shallow acetabular roofs. No acute fractures or dislocations are identified.

A four-year-old child with no family history of skeletal dysplasia, diagnosed with rhizomelic CP, laryngomalacia, and mosaic Down syndrome, presented to the emergency department with a two-day history of postprandial abdominal distension, associated with abdominal pain and irritability. The patient had a gastrostomy in place for the past two years due to feeding difficulties related to underlying comorbidities; therefore, no recent surgical intervention had been performed prior to symptom onset. Physical examination revealed a gastrostomy site with fecaloid biliary discharge, a distended and tympanic abdomen, and an abdominal circumference of 45 cm. Laboratory tests were within normal limits. Given the clinical presentation, intestinal obstruction versus paralytic ileus was suspected. An abdominal and chest X-ray was obtained, with the upper limbs demonstrating shortening (white arrows) with punctate calcifications in the cartilaginous regions (Figure 1). Additionally, to confirm, X-rays of the spine, humerus, and pelvis were requested, revealing findings of sclerotic bone lesions, shortening, and punctate calcifications in the cartilage with epiphyseal and metaphyseal anomalies (Figure 2). 

A multidisciplinary meeting with pediatric surgery was conducted. Based on imaging and clinical findings, there was marked bowel loop distension and colonic dilation due to intestinal malrotation and internal hernia through a mesenteric defect, for which a surgical approach was indicated. The patient required lysis of peritoneal adhesions and fibrous bands and antegrade decompression of the small intestine and colon. Postoperatively, the patient demonstrated an adequate initial response and recovery, with a clean surgical wound and no leakage or bleeding. However, on postoperative day four, the patient developed a fever and moderate bilious drainage, which led to the initiation of parenteral nutritional support along with glutamine and thiamine supplementation due to the risk of refeeding syndrome.

Subsequent laboratory and blood culture tests revealed normocytic anemia, elevated C-reactive protein, hypocalcemia, and hyperchloremia, with normal renal function and negative blood cultures at 72 hours. In the following days, the patient presented with renewed symptoms of bowel obstruction. Empiric antibiotic therapy was initiated due to suspected bacterial infection, and surgical reintervention was required due to the volvulus of the terminal ileum.

Following the procedure, the patient experienced gradual clinical improvement. At follow-up, caregivers were instructed on the need for oral suctioning in the presence of secretions. A rehabilitation plan was also established, focused on enhancing swallowing function and providing perioral and intraoral sensory stimulation.

Early diagnosis of CP is essential to optimize patient outcomes and prevent avoidable complications, as the patient presented. Therefore, recognition of typical radiologic findings, including epiphyseal stippling in the immediate postnatal period, clinical features like rhizomelic shortening and nasal hypoplasia, and genetic and metabolic studies play a pivotal role in accurately discerning these conditions. Multidisciplinary management initiated early in life can address orthopedic, respiratory, and feeding issues before they become severe. Preventive strategies include close developmental monitoring, proactive nutritional support, and early rehabilitation interventions. 

## Discussion

CP is a rare entity defined as a clinically heterogeneous group of skeletal and cartilage dysplasias associated with epiphyseal stippling and cervical instability that leads to neurological deficits, and in which arylsulfatase E (ARSE) mutations have been identified in only 50% of male patients [7, 8, 2]. The pathogenesis is based on a peroxisomal, X-linked disorder (CPX1) secondary to mutations in the ARSE gene [9], and other cases in the literature described that were not associated with peroxisomal dysfunction. These cases were found to be secondary to teratogen exposure or maternal conditions [10].

Although CP has been associated with a wide range of genetic and metabolic conditions, its coexistence with Down syndrome has been documented only rarely. The literature is limited to isolated case reports, without a clear pathogenic mechanism explaining the association. Some authors have suggested that the occurrence may be incidental, while others have speculated about shared developmental pathways contributing to skeletal abnormalities. The presence of Down syndrome in our patient further complicates the phenotypic expression, with overlapping features such as growth restriction and dysmorphic facial characteristics. Recognizing this unusual association is important for radiologists and clinicians, as it extends the differential diagnosis of skeletal dysplasias in children with chromosomal abnormalities.

Clinical features such as nasal hypoplasia, disproportionate dwarfism and bony abnormalities, severe mental retardation, joint contractures, cataracts, recurrent respiratory infections, and breathing problems [11]. Since it is uncommon, the diagnosis poses a challenge in CP due to requiring an early diagnosis based on recognition of clinical exam, radiographic findings, and molecular findings [12]. Although it requires a hemizygous ARSL pathogenic variant by molecular genetic testing, this exam is not available on a clinical basis [11]. Laboratory tests that may reveal associated metabolic abnormalities [2]. In this case, the symptoms of the patient with abdominal distension and fecaloid discharge were presented secondary to intestinal obstruction associated with postoperative complications of the gastrostomy and were not directly caused by the CP itself. However, it is important to mention that the underlying skeletal dysplasia and associated systemic comorbidities, including Down syndrome, may have contributed to the patient’s overall susceptibility and increased risk of complex clinical presentations.

In our case, the child initially presented with an acute abdominal emergency, characterized by abdominal distension and suspected intestinal obstruction. Although gastrointestinal manifestations are not a typical feature of CP, children with multisystem genetic disorders, including skeletal dysplasias, may present with acute abdominal conditions that complicate the clinical condition. Differential diagnoses in this case include paralytic ileus secondary to respiratory compromise, adhesions related to previous gastrostomy, or, less frequently, intrinsic intestinal anomalies.

Imaging modalities such as radiographs, ultrasound (US), and computed tomography (CT) are essential to confirm the presence of epiphyseal stippling, to assess the severity of skeletal abnormalities and the characteristic stippled calcifications of unossified cartilaginous epiphyseal centers during the first year of life visible on radiographs, and to exclude other differential diagnoses such as other skeletal dysplasias [3,13,14]. 

Antenatal diagnosis can be made through sonography, which depends on recognition of the combination of rhizomelic bone shortening and punctate epiphyseal calcifications, and family history of the disease. The typical findings are a pattern of bone shortening, profound hypoplasia of the humeri, flaring of the metaphysis, and disordered premature calcifications of epiphyses [14]. 

The prognosis is poor due to the correlation between clinical severity and the effect of the mutation on PEX protein function to eventual death [4,8]. Treatment is not definitive, although management protocols have been suggested [4].

## Conclusions

CP is a rare skeletal dysplasia that presents diagnostic and therapeutic challenges. The limited number of reported cases in the literature, combined with their variable phenotypic expression and overlapping radiologic features with other conditions, makes early recognition difficult. For this reason, radiologists' and clinicians' awareness of the characteristic imaging findings, particularly epiphyseal stippling and rhizomelic limb shortening, is essential to guide timely diagnosis and appropriate multidisciplinary management.

Given that CP is an entity that requires prenatal diagnosis, it is important to emphasize the role of fetal sonography in early detection. Prenatal diagnosis allows for timely counseling of parents, recognizes complications, and allows for the evaluation of multidisciplinary care from birth. Publishing additional cases to ours is crucial to awareness among radiologists and sonographers to ensure that such rare entities are recognized during the prenatal period whenever possible.
